# Anesthetic management of epilepsy surgery in a patient previously diagnosed with Takotsubo cardiomyopathy: A case report

**DOI:** 10.1097/MD.0000000000031229

**Published:** 2022-10-21

**Authors:** Yue Xu, Yi Li, Jinhua He, Jianli Li, Meinv Liu, Huanhuan Zhang

**Affiliations:** a Department of Anesthesiology, Hebei General Hospital, Shijiazhuang, China.

**Keywords:** anesthetic management, epilepsy, Takotsubo cardiomyopathy

## Abstract

Takotsubo cardiomyopathy (TC), also known as stress cardiomyopathy, apical ballooning syndrome, octopus pot cardiomyopathy and broken heart syndrome, is characterized by wall motion abnormalities of the left ventricle.

**Patient concerns:**

Here, we reported a 73-year-old woman diagnosed with TC induced by epilepsy before 3 months presented to the authors’ hospital with generalized tonic-clonic seizure. She was scheduled for intracranial tumor resection to cure the epilepsy.

**Diagnosis:**

She was diagnosed with epilepsy and TC. Interventions: Anesthesia management plays an important role in patients with a past history of TC.

**Outcomes:**

At the 1-week follow-up, she had fully recovered without obvious abnormalities.

**Lessons subsections:**

We emphasize the importance of individualized anesthesia management in patients with a past history of TC.

## 1. Introduction

Takotsubo cardiomyopathy (TC) is an acute reversible heart failure syndrome typically triggered by stressful events, which is much more common among females than males, especially in older adults.^[1]^ Typically, the temporary decline in ejection fraction resolves within a few days to weeks. TC might develop when sympathetic nervous system was hyperactivated in stressful conditions including seizure. Recent study suggests that 1 in every 1000 epilepsy-related hospitalizations might occur secondary TC,^[2]^ which can be a serious complication of seizures. In this case, we present the anesthesia management of epilepsy surgery in a female patient previously diagnosed with TC.

## 2. Case presentation

A 73-year-old woman (weight: 50 kg, height: 160 cm) with a history of diabetes, symptomatic epilepsy and TC, experienced a generalized tonic-clonic seizure, was admitted to the authors’ hospital. She was scheduled for epilepsy surgery (intracranial tumor resection) under general anesthesia.

**Figure 1.**
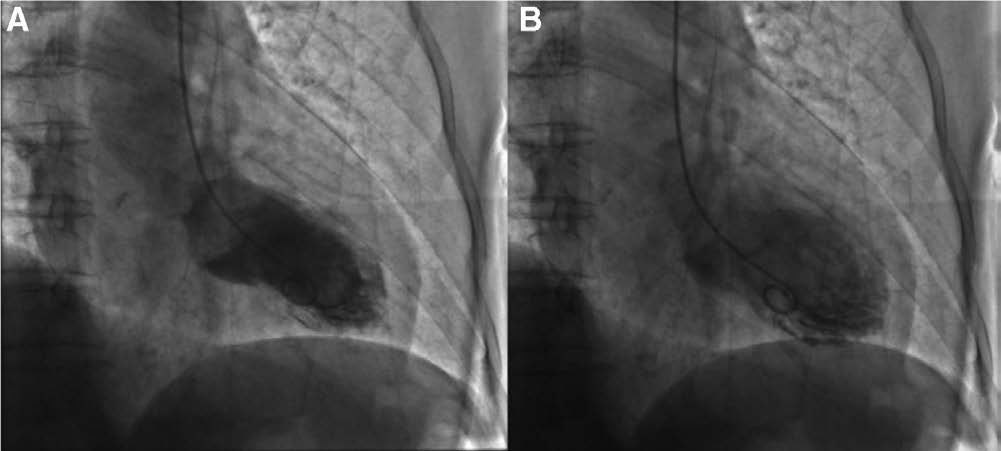
The left ventriculogram (A and B) showed the characteristic apical ballooning and hypercontraction of the basal segments.

**Figure 2.**
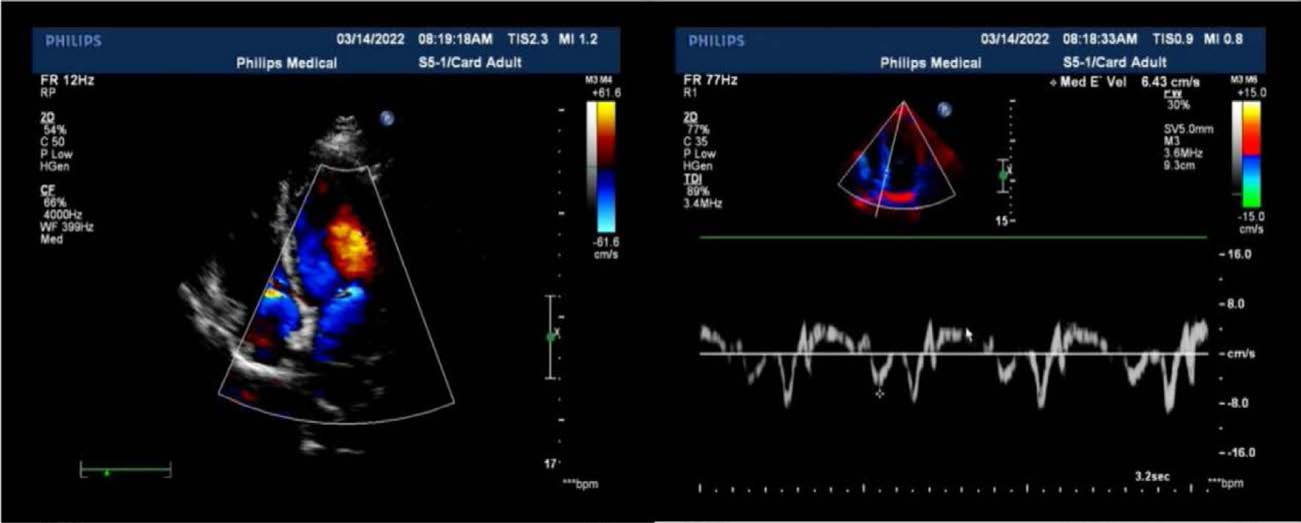
Transthoracic echocardiography after resolution of TC. No obvious abnormality of wall motion is visible. Aortic valve regurgitation was minimal. TC: Takotsubo cardiomyopathy.

**Figure 3.**
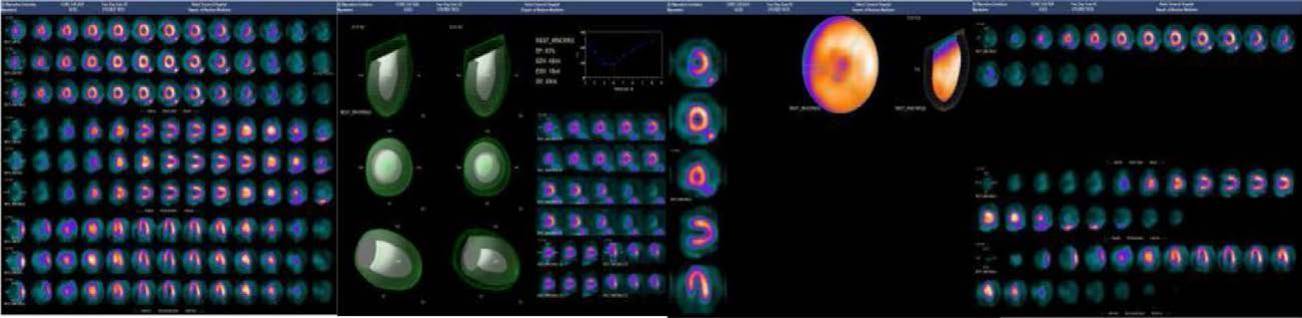
Myocardial perfusion imaging showed no apparent myocardial damage.

She was diagnosed with epilepsy 3 months prior to this attack. Her seizures were described as being generalized tonic-clonic, accompanied by severe chest pain, dyspnea, diaphoresis, and nausea. Laboratory examinations showed noticeable abnormalities in troponin-T: 0.152 ng/mL and B-type natriuretic peptide: 3499 pg/mL. Transthoracic echocardiogram revealed evidence of moderate left ventricle systolic dysfunction (ejection fraction 31%) and akinesia of the left ventricular anterior wall to the apex. Coronary angiography showed normal coronary anatomy without obstructive lesions. Moreover, the left ventriculogram showed apical ballooning, which was consistent with TC (Fig. 1). She had no family history of cardiopulmonary disease. The patient was then transferred to the coronary care unit and received the following treatments: aspirin 100 mg daily, atorvastatin 20 mg daily, spironolactone 20 mg daily, and rabeprazole 20 mg daily through oral administration, with 4000 IU of subcutaneous lowmolecular-weight heparin every 12 hours and continuous intravenous pumping of recombinant human brain natriuretic peptide 0.5 mg. The clinical and hemodynamic conditions gradually improved 4 days later. Repeat echocardiography revealed normal heart function with an Left Ventricular Ejection Fraction of 72% (Fig. 2). Myocardial perfusion imaging showed no obvious abnormalities (Left Ventricular Ejection Fraction: approximately 63%) (Fig. 3). The patient was discharged home with aspirin, ivabradine hydrochloride, spironolactone, and metoprolol tartrate.

She was readmitted to the authors’ hospital for intracranial tumor resection to cure the epilepsy 3 months after discharge. Upon admission, the results of preoperative evaluations (pulmonary and airway examinations) were unremarkable. Electrocardiography showed T wave inversions. No abnormalities of laboratory values were found. Hence, her American Society of Anesthesiologist score was II. We applied the estazolam 1 mg through oral administration for preoperative sedation at the night before the surgery. In the operating room, intraoperative monitoring included 5-lead electrocardiography, invasive blood pressure, blood oxygen saturation, end-tidal carbon dioxide, nasopharyngeal temperature, bispectral Idex, cardiac output and urine output. Blood gas analysis before surgery showed the following: pH: 7.50, PaO_2_ 74 mm Hg, PaCO_2_ 43 mm Hg, Lac 0.7 mmol/L, Glu 11.0 mmol/L, K^ + ^3.5 mmol/L, BE 9.3 mmol/L. Prior to anesthesia induction, the patients received 5 mL/kg of lactated Ringer solution. A loading dose of dexmedetomidine (1 µg/kg) was administered for 15 minute intravenously. Afterwards, uneventful rapidsequence induction of general anesthesia was performed, including sufentanyl 1 μg/kg intravenous, etomidate 0.4 mg/kg, and rocuronium 0.9 mg/kg, respectively. The patient was received 2% lidocaine 3 mL, which was sprayed on the tongue base, epiglottis, and glottis to achieve uniform surface anesthesia before endotracheal intubation. The anesthesia was maintained with propofol (2–4 mg·kg^–1^·h^–1^), remifentanil (0.1–0.3 μg·kg^–1^·h^–1^), sevoflurane (0.5%–1%) and dexmedetomidine (0.4–0.6 μg·kg^–1^·h^–1^). Intraoperatively, the patient’s vital signs were stable with blood pressure maintained at 130 to 150/65 to 75 mm Hg and heart rate of 55 to 65 beats/minute. The operation lasted for 2 hours and proceeded smoothly. Then she was transferred to Postanesthesia care unit with the maintenance infusion of dexmedetomidine (0.4 μg·kg^–1^·h^–1^). The tracheal tube was removed after the patient was fully awake without obvious hemodynamic fluctuations, and dexmedetomidine infusion was stopped. Blood gas analysis at Postanesthesia care unit showed the following: pH: 7.47, PaO_2_ 110 mm Hg, PaCO_2_ 42 mm Hg, Lac 0.7 mmol/L, Glu 9.1 mmol/L, K + 3.7 mmol/L, BE 6.3 mmol/L. She was then transferred to the ward with a heart rate of 65 beats/minute, blood pressure of 145/75 mm Hg, and SpO_2_ of 100% (oxygen flow of 2 L/minute through a nasal cannula). The patient was discharged 13 days after surgery and did not experience postoperative complications. At the 1-week follow-up, she had fully recovered without obvious abnormalities.

## 3. Discussion

TC, also known as stress cardiomyopathy, apical ballooning syndrome, octopus pot cardiomyopathy, and broken heart syndrome, is characterized by wall motion abnormalities of the left ventricle. TC is usually triggered by a stressful situation either physical or emotional. Neuro-cardiogenic interaction has been known for many years.^[3]^ Various neurological conditions are associated with TC include stroke, transient ischemic attack, subarachnoid hemorrhages and seizures.^[2]^

Cerebral seizure has been reported as a cause of TC internationally, and cardiac complications are 1 of the main causes of mortality in epilepsy.^[3]^ Published reports of TC existed in various surgical specialities including general surgery, orthopedics, gynecology and neurosurgery.^[4]^ An excessive release of catecholamines, known as a trigger of TC, is developed when sympathetic nervous system is hyperactivated in stressful conditions such as cerebral seizure.^[5]^ We report a 73-year-old woman who was previously diagnosed with TC caused by epilepsy and scheduled for epilepsy surgery. A recently published systematic review of TC cases reported that surgery acts as psychologically and emotionally taxing.^[4]^ In many TC cases, patient prognosis is good, while it had a propensity for recurrence and recurrence rate was approximately 8%.^[6]^ Therefore, how to appropriately control the stress response to prevent recurrence during the perioperative period poses a great challenge for anesthesiologists.

We successfully performed general anesthesia in a patient previously diagnosed with TC result from epilepsy undergoing intracranial tumor resection. Throughout the perioperative period, the anesthesiologist should remain vigilant as to the possible development of hyperadrenergic states such as subarachnoid hemorrhage and epilepsy which might induce TC attack. There is no established guideline for the anesthetic strategy to prevent recurrence of TC in patients with a prior history of TC.^[6]^ A previous case report suggested that a deeper level of anxiolysis before the operating room might be beneficial.^[6]^ In our patient, estazolam was used for sedation, anti-anxiety and sleep improvement the night before the surgery. Moreover, airway manipulation was as brief and gentle as possible to avoid sympathetic nerve stimulation and excessive release of catecholamines. Topical airway anesthesia was performed prior to endotracheal intubation. Induction and maintenance anesthetics were selected with the lowest probability of myocardial inhibition to prevent hemodynamic instability. Remifentanil significantly suppresses the secretion of epinephrine and norepinephrin,^[7]^ so it was used for intraoperative analgesia in our patient. Moreover, it is crucial to measure fluid status and avoid volume overload. Stroke volume variation-guided goal-directed fluid therapy was used to keep the optimal volume state, maintaining stroke volume variation  < 13%. Hypothermia, a trigger for stress, is common under anesthesia. Maintaining normothermia can mitigate the degree of stress and the clinical risks.^[8]^ In our patient, insulation blanket was used to provide additional thermal protection (temperature: 36.5°C) to reduce the occurrence of perioperative hypothermia. Successful hemodynamic management should exists in equilibrium with surgical stress. Our patient didn’t experienced significant hemodynamic fluctuations in the perioperative period. In a word, minimal stimulation and careful monitoring throughout the perioperative period for early diagnosis of possible acute complications seems to be the safest option.

Evidence-based guidelines for the management of TC are lacking, treatment relies on supportive treatment and maintaining hemodynamic stability. Centrally acting alpha-2 agonists such as dexmedetomidine and clonidine have also been considered in the treatment of TC.^[5]^ By reducing sympathetic outflow, these drugs may be beneficial in preventing recurrence in at-risk patients, particularly in the perioperative setting. In our case, the patient experienced less pain along with lower analgesic requirements due to receiving dexmedetomidine. Thoracic epidural anesthesia extending to the upper thoracic region (T1 to T5) induces cardiac sympatholysis and may be ideal for reducing sympathetic outflow in patients with a history of TC who are undergoing abdominal or thoracic procedures.^[9]^ In an experimental model of TC, isoflurane, but not propofol, exerts a cardioprotective effect.^[10]^

In conclusion, we describe the anesthesia management of epilepsy surgery in a patient previously diagnosed with TC and epilepsy. This case highlights that patients diagnosed with TC and undergoing surgery needs an individualized anesthesia approach to avoid possible recurrence or fatal consequences. It is hoped that our case could provide experience for anesthesia management in patients with TC.

## Author contributions

Yue Xu and Yi Li designed the study and approved the final manuscript. Jinhua He and Jianli Li designed the study and wrote the manuscript. Meinv Liu and Huanhuan Zhang wrote and edited the manuscript.

**Writing – original draft:** Yue Xu, Yi Li.

**Writing – review & editing:** Jinhua He, Jianli Li, Meinv Liu, Huanhuan Zhang.

## Acknowledgements

The authors thank this patient for providing this medical information.
